# Recognition and normalization of multilingual symptom entities using in-domain-adapted BERT models and classification layers

**DOI:** 10.1093/database/baae087

**Published:** 2024-08-28

**Authors:** Fernando Gallego, Francisco J Veredas

**Affiliations:** Departamento de Lenguajes y Ciencias de la Computación, Universidad de Málaga, Blvr. Louis Pasteur, 35, Puerto de la Torre, Málaga 29071, Spain; Research Institute of Multilingual Language Technologies, Universidad de Málaga, Facultad de Filosofía y Letras, Campus de Teatinos, Málaga 29071, Spain; Departamento de Lenguajes y Ciencias de la Computación, Universidad de Málaga, Blvr. Louis Pasteur, 35, Puerto de la Torre, Málaga 29071, Spain; Research Institute of Multilingual Language Technologies, Universidad de Málaga, Facultad de Filosofía y Letras, Campus de Teatinos, Málaga 29071, Spain

## Abstract

Due to the scarcity of available annotations in the biomedical domain, clinical natural language processing poses a substantial challenge, especially when applied to low-resource languages. This paper presents our contributions for the detection and normalization of clinical entities corresponding to symptoms, signs, and findings present in multilingual clinical texts. For this purpose, the three subtasks proposed in the SympTEMIST shared task of the Biocreative VIII conference have been addressed. For Subtask 1—named entity recognition in a Spanish corpus—an approach focused on BERT-based model assemblies pretrained on a proprietary oncology corpus was followed. Subtasks 2 and 3 of SympTEMIST address named entity linking (NEL) in Spanish and multilingual corpora, respectively. Our approach to these subtasks followed a classification strategy that starts from a bi-encoder trained by contrastive learning, for which several SapBERT-like models are explored. To apply this NEL approach to different languages, we have trained these models by leveraging the knowledge base of domain-specific medical concepts in Spanish supplied by the organizers, which we have translated into the other languages of interest by using machine translation tools. The results obtained in the three subtasks establish a new state of the art. Thus, for Subtask 1 we obtain precision results of 0.804, F1-score of 0.748, and recall of 0.699. For Subtask 2, we obtain performance gains of up to 5.5% in top-1 accuracy when the trained bi-encoder is followed by a WNT-softmax classification layer that is initialized with the mean of the embeddings of a subset of SNOMED-CT terms. For Subtask 3, the differences are even more pronounced, and our multilingual bi-encoder outperforms the other models analyzed in all languages except Swedish when combined with a WNT-softmax classification layer. Thus, the improvements in top-1 accuracy over the best bi-encoder model alone are 13% for Portuguese and 13.26% for Swedish.

**Database URL**: https://doi.org/10.1093/database/baae087

## Introduction

The digital age is continuously redefining the boundaries of the medical sector, particularly with the growing adoption of electronic health records (EHRs). These documents represent an evolution from traditional clinical records, enhancing efficiency in information extraction and facilitating a deep and detailed understanding of patient health profiles. This is not merely a technical change but also marks a turning point in health management and analysis, as it is crucial for improving patient care, allows for storage, and represents a transition toward large, structured, and analyzable knowledge bases (KBs).

As this transition progresses in the biomedical domain, the amount of data present in these records significantly increases, requiring sophisticated methods for the correct extraction of information and its subsequent use. In response to this major challenge, integrating techniques such as natural language processing (NLP) into clinical records management is an efficient solution that has opened new avenues for patient care and the analysis of large volumes of biomedical data.

Within this context, named entity recognition (NER), named entity linking (NEL) and the resulting normalization emerge as critical solutions for effectively managing the information contained within medical data, facilitating its interpretation and practical use. While NER aids in identifying medical terms within unstructured data, NEL aims at linking these concepts to a standardized and normalized vocabulary. This process represents a further step toward automatic coding, improving the information extracted from these types of documents and thereby positively impacting any subsequent analytical or research approach based on this information.

However, the use of NLP in clinical texts faces significant challenges, such as semantic ambiguity and linguistic variability, which makes it difficult to accurately identify clinical entities and match them with unified standards, affecting the reliability of decisions made from this information. These difficulties have led NLP experts to enhance the entire process, from developing more sophisticated models for the recognition of medical entities to implementing methods for their proper classification and association with standardized codes.

Technical advances in recent years have successfully established a solid foundation for entity identification in multilingual clinical texts, while the next natural step is to accurately link these entities to major medical standards such as UMLS (Unified Medical Language System) [1] or SNOMED CT (Systematized Nomenclature of Medicine Clinical Terms) [2]. These data corpora contain a vast amount of information, with UMLS alone comprising over 4.5 million concepts. However, navigating these databases can be complex. Due to the nature of these KBs, a single concept might be annotated with multiple codes from different categories or, because of the graph structure of ontologies, repeated concepts may be encountered when exploring their hierarchical levels.

In this paper, we present the complete pipeline, from the recognition of clinical entities to their linkage to a knowledge base such as SNOMED CT. We pay special attention to multilingual linking and highlight the challenge of dealing with under-resourced languages, which consequently have less presence in current databases, by means of machine translation.

## Related work

Once medical records have been digitized into EHRs, extracting and leveraging the information contained in these records is one of the main challenges encountered by NLP tools. With the aim of advancing the state of the art of biomedical text processing techniques, numerous shared tasks have been proposed in recent years. Some of these shared tasks involve evaluation campaigns aimed at the creation of tools and models oriented to specific tasks, such as the recognition and standardization of medical entities named in texts written in a given language, such as Spanish. Along these lines, within the PlanTL of the Spanish government, a series of shared tasks has been proposed for progressing in the field of biomedical text mining in Spanish, such as DisTEMIST [3], MedProcNER [4] or, more recently, SympTEMIST [5]. The SympTEMIST shared task was undertaken by the Barcelona Supercomputing Center (BSC) and designed to improve the detection and normalization of symptoms, signs, and findings in medical documents in Spanish. This task involved three subtasks: Subtask 1, called *SymptomNER*, focused on the recognition of symptom, sign, and finding entities in clinical texts; Subtasks 2 and 3, named *SymptomNorm* and *SymptomMultiNorm*, respectively, were dedicated to the designing of systems to match these named entities to concepts of the SNOMED-CT ontology, both in Spanish and in other proposed languages (namely, Catalan, Dutch, English, French, Italian, Portuguese, Romanian, and Swedish).

Despite the challenges posed by the complexity of biomedical texts, recent advancements in machine learning, particularly in Transformer-based models, offer promising solutions for entity recognition tasks. These innovative techniques underscore the importance of accurately detecting and normalizing medical entities as a foundational step in biomedical text processing.

### Named entity recognition

To address the complete pipeline for the detection and subsequent normalization of entities, the first step is to accurately recognize the entities present in the biomedical texts to be analyzed. In this regard, the dominant trend in NER over the last few years has been the use of Transformer-based models, due to their high performance in dealing with language comprehension tasks. Thus, López and coauthors [6] leveraged a transfer-learning strategy to firstly adapt a BERT-based multilingual model to the clinical-oncology domain in Spanish—by continuously pretraining the language model on a proprietary corpus called Galén [7]— and then fine-tune the resulting model to address clinical-coding downstream tasks. Transfer learning is among the most prevalent methodologies in NER, demonstrating that continual pretraining and domain adaptation of large language models (LLM) leads to improved performance in these tasks. The approach followed in this study for medical NER originates from these transfer-learning strategies. Another emerging research direction is the application of generative models to NER tasks, where the aim is to design a series of templates or prompts that feed the system in such a way that they can extract meaningful information—recognizing entities from a text, linking these entities to a standardized ontology, etc. Following this approach, Cui and collaborators [8] proposed a series of templates designed to adapt a BART [9] model to zero or few-shot scenarios for NER. Although the use of generative models sounds very promising, it is not yet common in the medical domain due to the complexity of the biomedical texts—in terms of the number of existing medical concepts, the extensive use of abbreviations, the presence of synonyms and ambiguous terms, and heterogeneity in general.

### Named entity linking

Once the biomedical entities present in a clinical text have been obtained, the next step is to normalize these entities to the concepts of an existing standardized vocabulary or ontology. Unlike NER approaches, classification is not usually one of the most used methodologies for NEL due to the scarcity of annotated data needed for training, therefore resulting in numerous zero- or few-shot scenarios in inference time. For this reason, one of the main strategies that contribute to the successful of these methods is enrichment of biomedical corpora—used to train NEL models—with data from in-domain KBs like SNOMED-CT or UMLS. This approach strives to mitigate the apparent limitations of language models by incorporating these KBs for enhanced adaptation of the NEL models to the clinical domain [10]. This methodology is notable for supplying the models with improved capacity to comprehend and process the complexities and nuances of medical texts, while achieving usually earlier convergence. However, securing KBs that contain quality data can be a challenging task, as many of these are not readily accessible or are biased. In our case, we count on the advantage of having access to a KB relevant to the task, as the SympTEMIST organizers supplied a gazetteer that included all terms (each one associated with a unique concept code from SNOMED-CT) involved in the normalization procedure.

In the case of NEL, the combination of generative AI and prompt-learning strategies is also present in the literature, largely due to the recent results achieved by LLMs in numerous biomedical NLP tasks, which have propelled their integration into the medical field. Thus, the work of Yuan and coauthors [11] emphasized the performance of these LLMs in the biomedical domain. After fine-tuning the BART model [9] with PubMed abstracts, the authors showcased the model’s efficacy in tackling diverse biomedical text generation tasks, which opens the possibility of using prompt-engineering techniques to adjust these models and obtain desired results, such as recognized and standardized entities. Also recently, Dig and collaborators [12] capitalized on the impressive performance of LLMs to apply prompt learning strategies to fine-tuning these models for NEL tasks. Likewise, Yuan and coauthors [13] succeeded in harnessing the functionalities of both approaches—prompt learning and generative AI—by presenting a generative LLM trained with synthetic samples with synonyms and definitions from KBs. Again, the main limitation of these approaches is access to quality data, in addition to the suboptimal performance achieved by the LLMs in NEL tasks where samples are scarce (few-shot scenarios) or even not available (zero-shot scenarios). Although this approach sounds promising, there is insufficient information to determine if it is the optimal development methodology to be adopted for biomedical NEL in the near future.

With the specific objective of recognizing sign, finding or symptom entities in clinical texts, Jonker and coauthors [14] participated in SympTEMIST shared task by developing a system called SymptomNER, which integrates a RoBERTa Transformer model, complemented with a BiLSTM recurrent network and a masked conditional random field layer, to tackle NER of symptoms as a classification task. Moreover, the authors used a data augmentation strategy during the training phase to enhance the model’s robustness and generalization capabilities. Their methodology is particularly relevant to our study given that—like our approach—Jonker and collaborators chose to split the input texts into text chunks to optimize the system’s capability to deal with long sequences of tokens. Whereas in their strategy the input texts were divided into batches of a maximum of 512 tokens, in our study we have preferred division based on complete sentences to facilitate contextual interpretation of the text by the model. In a complementary line of research, Borchert and collaborators [15] explored a similar approach consisting in using Transformer-based models for the recognition of symptom entities in clinical notes, employing RoBERTa as the core of their system for NER. Parallel to our approach, they adopted a sentence-based text segmentation strategy, using the spaCy tool (https://spacy.io/) for this purpose. This aspect is particularly relevant, as their model is adapted to the morphosyntactic particularities of the Spanish language, which underscores the importance of considering linguistic specificities in NLP task. In contrast, our study focuses on a preprocessing stage which, although also based on sentence segmentation, is specifically designed to optimize the identification and handling of punctuation marks.

In the NEL context of SympTEMIST shared task, Borchert and collaborators’ work with the xMEN framework [16], based on SapBERT [17], stands out for its cross-lingual ability to link medical entities. Their method excelled in initial tests, setting a milestone in precision for the analysis of clinical texts. On the other hand, Grazhdanski and coauthors [18] made advances in the linking of medical entities using SapBERT-XLMR-large, adopting a methodology that included data enrichment through UMLS synonyms for the concepts present in a specialized gazetteer. This approach was enhanced using cosine similarity to assess the relationship between each mention and the concepts in their KB, selecting the concept with the highest similarity score.

Finally, an encouraging trend, and the focus of this paper, is the use of classification-based approaches for biomedical NEL [19]. Such approaches have traditionally shown very high performance for all types of classification tasks, but in turn have presented significant limitations in classification problems where there are both a high number of classes and a small number of training samples for the different classes. However, in this paper we show that it is possible to follow a classification approach to tackle a NEL task in which the number of classes—that is, the number of unique concept identifiers—is on the order of hundreds of thousands. To this end, as discussed in the following sections, we employ a methodology that starts from embeddings provided by a Transformer-based model that has been pretrained to align medical entities in a continuous space formed by UMLS multilingual concept representations. These embeddings serve as initialization for an NT-softmax-like [19] classification layer that, once it has been fine-tuned on a given training set, allows to obtain high performance in the normalization of entities in various languages, while reducing the computational cost that could result from the need to retrain language models in specific languages.

This solution accesses each of the descriptions and synonyms provided for a given dataset through a gazetteer or KB, achieving high performance as it will be shown in the following sections. This methodology is applicable to any language for which this series of annotations is available, without the high computational requirements of current bi-encoder and cross-encoder training methods.

## Materials and methods

The following sections outline the corpora used for each of the SympTEMIST subtasks, along with the methodology employed for each and the approach taken to address them. For the reproducibility of all the experiments conducted in this study and the access to our proposed NER + NEL pipeline, the source code and the necessary datasets have been uploaded to a public repository at GitHub (https://github.com/ICB-UMA/WNT-Softmax.git).

### Description of the Corpora

**Figure 1.**
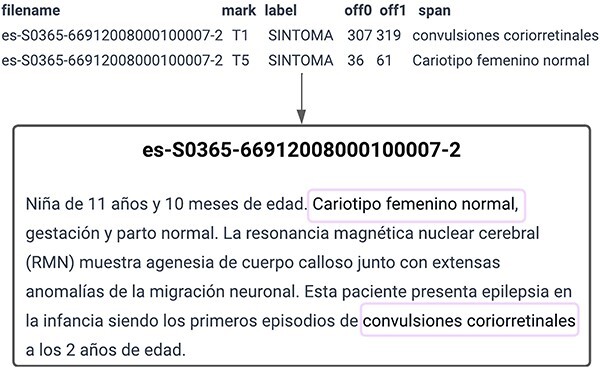
SymptomNER: Symptoms, signs and findings Named Entity Recognition subtask.

The organizers of the SympTEMIST shared task provided participants with a corpus consisting of 1000 clinical cases with annotations of symptoms, signs, and findings normalized to SNOMED CT. This corpus was meant to be used to solve three different tasks: SymptomNER (Subtask 1), for the recognition of clinical entities in Spanish, SymptomNorm (Subtask 2) and SymptomMultiNorm (Subtask 3), for the normalization of clinical named entities in Spanish and multilingual scenarios, respectively. Figure 1 shows a sample provided for Subtask 1, SymptomNER. It includes the text from one of the training set documents along with the annotations that the models are expected to learn to perform.

SympTEMIST makes available to researchers a medical corpus of clinical notes in Spanish, with 12 196 annotations made by clinicians and language experts. These clinical notes were distributed in a total of 750 training documents and 250 test documents (with 9092 annotations in the training set and 3104 in the test set), covering a wide range of medical specialties such as cardiology, oncology, rheumatology, dentistry, urology, and mental health. All the mentions present in these text documents were manually annotated—marking the given mention’s text-span (see Fig. 1)—while 6588 (3484 in training set and 3104 in the test set) of the annotated mentions were also labeled by mapping them to the corresponding SNOMED-CT concept identifier. For Subtask 1, we initially decided to divide the documents into a training set and a validation set and allocate one-third of the annotations in the validation set. However, with the aim of ensuring that all the annotations from a single document stayed in the same subset, whether for training or validation, the training set consisted finally of 520 documents with 6354 annotations, while the validation set included 224 documents with 2738 annotations (6 out of the 750 clinical notes missed normalization annotations). Additionally, the number of sentences per document was analyzed to ensure that the length of both sets was roughly similar, confirming that this trend was maintained (Table 1).

**Table 1.** Dataset division for Subtask 1, detailing annotations (anns), documents (docs), average sentences per document (sent/doc), and average tokens per document (tokens/doc) for training and validation sets

Dataset	anns	docs	sent/doc	tokens/doc
Train	6354	520	15.88	1235
Validation	2738	224	15.93	538
Total	9092	744	15.91	1773

In the effort to normalize medical entities in Spanish for Subtask 2, SymptomNorm, which focuses on symptom normalization and entity linking, the organizers of the shared task provided us with a training dataset comprising 3484 normalized mentions. It is important to highlight that 59 of these entities lacked assigned codes, leading to their exclusion in the analysis. The procedure followed to maximize the outcome on the test set involved splitting this training set, reserving a portion for validation (25%), stratifying by code, and, for cases with only a single entry, keeping it within the training set. Consequently, our validation set contains 729 records. Moreover, for fine-tuning the WNT-softmax classification layer, we have concatenated the resulting training set with all the entries extracted from the specialized gazetteer supplied by the organizers (see Table 2, first row). This gazetteer is composed of a subset of clinical terms drawn from SNOMED-CT that consists of fully specified names and synonyms relevant to the NEL task and associated with the corresponding medical concept (with unique code). Moreover, each entity mention/term (e.g. “neoplasia de pulmón”) in this resulting training set was enhanced with its corresponding semantic tag (e.g. “finding”) extracted from the given gazetteer to enrich the clinical term with information that facilitates the matching of entities to their corresponding concepts (with associated concept unique identifier/code) in inference time. This way, each entry in the final training set has the form of “term [semantic_tag].”

**Table 2.** Multilingual entity linking: annotation distribution by language for training, validation, and testing

Language	Train	Validation	Test	UMLS concepts	UMLS %
Spanish	167 514	728	2848	1 371 261	10.08
Catalan	165 923	646	2523	0	0.00
Czech	161 367	126	561	211 039	1.55
English	162 459	428	1600	8 603 905	63.22
French	163 863	379	1425	430 407	3.16
Italian	163 362	408	1544	247 419	1.82
Dutch	163 053	339	1249	288 964	2.12
Portuguese	161 679	369	1521	427 425	3.14
Romanian	163 089	287	1065	0	0.00
Swedish	161 643	291	1118	155 391	1.14

The training sets include the integrated gazetteer. Additionally, for languages other than Spanish, machine translation has been performed.

Finally, in Subtask 3, SymptomMultiNorm, training data were provided by the organizers, separated by languages including Catalan, Czech, English, French, Italian, Dutch, Portuguese, Romanian, and Swedish. The approach we adopted closely mirrored that of Subtask 2, involving the division into training and validation sets, stratification by code, and integration of the gazetteer into the training set. A notable distinction for this subtask was that the gazetteer was exclusively in Spanish, motivating the use of the *MarianMT framework for translation* models (https://huggingface.co/docs/transformers/model_doc/marian), popular for their proficiency in machine translation, to tailor the content to each specific language. As in the case of Subtask 2, the entities of the training set consist of terms and their corresponding semantic tags. It is important to note that not all datasets are of the same size, although they do contain the translated gazetteer.

### Clinical entity recognition and linking in Spanish

The detection of medical entities in clinical notes and their subsequent linkage to different standardized dictionaries or ontologies is established as a key objective within clinical NLP. Named-entity detection can address tasks such as recognizing locations, names, or organizations in generic corpora or, in the medical field, symptoms, diseases, procedures, medications, etc. It is in this domain where the SymptomNER subtask emerged as a shared task with the purpose of encouraging the development of systems aimed to the precise identification and classification of symptoms within clinical narratives in Spanish. The goal of SymptomNER was to be able to have effective models that facilitate the extraction of vital medical information from unstructured fields of electronic records, thereby allowing enhanced analysis and comprehension of health-related information.

In this study, the potential of multilingual BERT-based models such as mBERT [20] or XLM-RoBERTa [21], known for their multilingual proficiency, is leveraged for medical NER in Spanish. As is well known, the process carried out to adapt these models to the different languages was by pretraining them using multilingual corpora. Moreover, the capabilities of other monolingual BERT-based models, such as RoBERTa [22] (pretrained on corpora in English) or BETO [23] (pretrained on corpora in Spanish), have been also tested in this study to analyze the performance of these models in the scope of clinical NER in Spanish, while comparing their efficacy both individually and as part of ensembles of models.

On the other hand, we also wanted to analyze the effectiveness of using language models adapted to the clinical domain in Spanish, based on the hypothesis that these models can adapt to the subtleties and particularities of the natural language of the clinical setting in that language more effectively than multilingual language models that are not adapted to the clinical domain. In this sense, we analyze the effectiveness of several models based on the Transformer architecture, such as XLMR-Galén and mBERT-Galén [6], which start from multilingual BERT-based models and are adapted to the domain by means of continual pretraining with an oncology corpus in Spanish. Finally, we also analyze here the effectiveness of the BSC-Bio-es [24] and RoBERTa-Base-Biomedical-es [25] models, two models based on the monolingual RoBERTa model, which were pretrained on Spanish biomedical corpora.

To train all these models above we used the datasets detailed in “Description of the Corpora” section. In a preprocessing stage, we broke down the documents into sentences, saving the start and end of each sentence to accurately locate the mention within the precise segment. Subsequently, we processed the texts using an IOB annotation for the different sets: training, validation, and testing. To reassemble words from subwords, the reverse process of the aforementioned technique was employed, facilitating the extraction of identified entities from IOB tags. Moreover, during the phase of hyperparameter optimization, batch sizes ranged from 8 to 64, with learning rates adjusted between 1e-05 and 5e-05, leveraging the RectifiedAdam optimizer for the optimization process.

Once the individual performance of each model has been evaluated, we explore their potential to improve results by amalgamating their predictions and applying a majority voting strategy for decision making. Thus, we introduced two ensemble setups; the first one included all five models, while the second one was comprised of the top three performers based on the results from the validation set, i.e. XLM-R-Galén, BSC-Bio-es, and Roberta-Base-Biomedical-es.

Having thus established a robust NER model, our effort extended toward improving the results obtained by the models that currently set the state of the art in Subtask 2, SymptomNorm, which focused on entity linking and normalization in Spanish. Our preliminary strategy for this task involved integrating bi-encoder-based models with FAISS [26], while also considering the application of cross-encoders. For this purpose, in that preliminary work we used the multilingual SapBERT model [17] to generate embeddings for the various terms present in the provided gazetteer and to retrieve the most similar ones. Additionally, we initially explored the use of cross-encoders for re-ranking the candidates (which resulted in unsatisfactory outcomes as it decreased the initial performance of the bi-encoders), and a final step in which priority was given if the entity was in the gazetteer over the model’s prediction. However, this last step also decreased performance when the entity was composite—i.e. it had more than one associated code—and the evaluation selected a different code from the first one existing in the vocabulary.

In this study, we build on these preliminary results obtained [26] and aim to both train our own bi-encoders—by using a SapBERT-like [17] contrastive learning strategy for the self-alignment of all concepts of the UMLS in the five most frequent languages, i.e. English, Spanish, French, Italian, and Portuguese—and to combine them with a classification approach. For this purpose, XLM-RoBERTa models are employed as core models for contrastive learning, with the base version of XLM-RoBERTa (137 m parameters) pretrained for three epochs and the large version of the model (355 m parameters) pretrained for only one epoch (due to the significant increment of the computational cost for XLM-RoBERTa-large to be trained on our shared setup consisting of 8 A100 GPUs). Because of their analogy with SapBERT, we named these new bi-encoders SapXLMR, and used the sufixes -large-5lang an -base-5lang to distinguish between the two different versions that we propose and analyze herein, thus having SapXLMR-large-5lang and SapXLMR-base-5lang, respectively.

The endeavor of training a SapBERT-like model from such core models has highlighted the significant computational and time requirements involved. This has prompted our search of a classification-based approach that can retain the self-alignment capability of the bi-encoders, or even improve upon it, to adapt these large pretrained models to a specific language or domain without retraining them. The main challenge, as noted, lies in the complexity of this task, which involves over 120 000 different concepts and only 3000–5000 annotated entities. This classification layer—which we have named Weighted-Normalized-Temperature Softmax (WNT-softmax) and is based on the work of Xu and Miller [19]—is trained from the embeddings of the concept mentions present in the training set and the clinical terms in the KB (gazetteer) provided by the SympTEMIST organizers. Thus, given a set of entity mentions $\mathcal{M} = \{ {m_1},{m_2}, \ldots ,{m_t}\} $ from clinical notes, and a set of concepts $\mathcal{C} = \left\{ {{c_1},{c_2}, \ldots ,{c_n}} \right\}$ from a given ontology (e.g. SNOMED-CT) to which these entity mentions have to be linked to, the WNT-softmax determines the probability of a given entity mention $m$ being mapped to concept ${c_i}$ as


(1) $$p({c_i}|m) = \frac{{exp\left( {\frac{{{s_i}}}{\tau }} \right)}}{{\sum_{j = 0}^{n} exp\left( {\frac{{{s_j}}}{\tau }} \right)}},$$

where ${s_i} = \cos \left( {{w_i},Emb\left( m \right)} \right)$ is the dot product between the l2-normalized weight vector ${w_i}$ and the embedding of the entity mention $m$ given by the bi-encoder, and $\tau $ is a temperature factor that regulates the concentration level of the distribution.

The “weighted” characteristic of this dense layer stems from its initialization process, which is not random or from scratch but is instead based on the averaged embeddings of all the terms associated with the same concept, which include those entity mentions in the training set as well as those clinical terms (fully identified names and synonyms) in the gazetteer that can be mapped to the same concept (with a unique identifier/code in the ontology). Thus, the initial weights ${W_i}$, for a given concept ${c_i}$ in the WNT-softmax layer are calculated as the average ${\mu_{c_{i}}}$ of the embeddings of the entity mentions and terms ${t^{{c_i}}}$ that are associated with concept ${c_i}$:


(2) $${\mu_{{c_i}}} = {\ }Av{g_{{t^{{c_i}}} \in Term\left( {{c_i}} \right)}}\left( {Emb\left( {{t^{{c_i}}}} \right)} \right),$$

where $Term\left( {{c_i}} \right)$ is all the terms (i.e. entity mentions in the training set and terms in the gazetteer) that can be mapped to the same concept ${c_i}$ associated to a given unique identifier in the ontology.

The training of this WNT-softmax involves fine-tuning the weights using the embeddings of the entity mentions and terms from the training set and the gazetteer along with their corresponding linked concepts. This approach stands out for not requiring large computational resources, unlike bi-encoders, and allows for the specific adjustment of these embeddings for each task.

**Figure 2.**
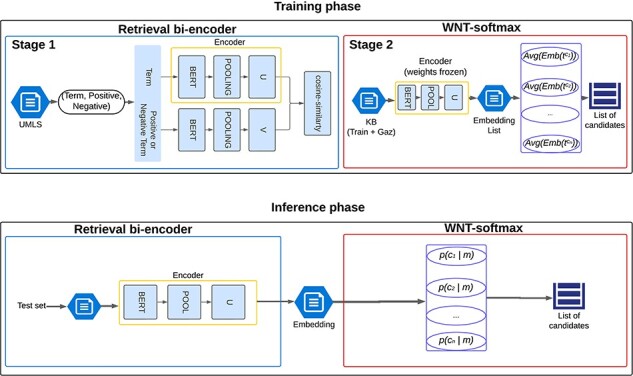
Illustration of the named-entity normalization process utilizing a pretrained bi-encoder architecture for efficient information retrieval, enhanced by the WNT-softmax layer for optimized classification and disambiguation.

To evaluate the efficacy of our classification-based approach, three different training runs of the WNT-softmax layer were conducted with each bi-encoder analyzed, varying the random seed to study the performance variability. However, as will be seen in “Results and Discussion” section, the difference between the results of the runs is minimal because the training datasets used are the same and the bi-encoders’ weights are frozen during training of the WNT-softmax. For the fine-tuning of this linear layer, the learning rate varied from 3e-05 to 5e-05, yielding very similar values. This entire procedure, including training and inference phases, is scheduled in Fig. 2.

### Standardization of multilingual clinical entities

Entity linking in multilingual medical texts presents significant challenges arising from linguistic diversity and the limited availability of annotated data. This difficulty is further amplified in a multilingual context, where annotations are even scarcer in less commonly used languages such as Czech or Romanian. For this reason, medical entity normalization models exhibit reduced accuracy in languages with sparse documents annotated, highlighting the urgent need for systems capable of adeptly managing this linguistic diversity.

A partial solution to this issue could be the use of bi-encoders based on multilingual LLMs, contrastively pretrained with UMLS terms, but, as mentioned above, they still have certain limitations and do not guarantee a good outcome for languages underrepresented in this ontology (e.g. Czech terms in the UMLS are only the 1% of the total). Moreover, if training one of these models for a specific language is already computationally expensive and time-consuming, training one for multiple languages significantly increases requirements in all aspects. These substantial limitations have underscored the development of new approaches that are more accessible for adaptation to such tasks without compromising performance.

Similar to the procedure followed for medical NEL in Spanish, we have evaluated both the performance of bi-encoders enriched with FAISS for candidate retrieval as well as in their integration with a classification strategy. Given that the provided gazetteer contains only concepts in Spanish, we have used automatic translation LLMs from the MarianMT framework. These translation models feature a generative architecture, specifically BART [9], and have proven to be highly effective in text translation tasks [27], a crucial aspect since clinical texts demand thorough terminological precision. Thanks to this approach, we have managed to generate over 160 000 training samples for each language (Table 2).

On the other hand, we have leveraged our access to the UMLS to pretrain our own bi-encoders, following a contrastive learning SapBERT-like strategy and starting from the base and large versions of XLM-RoBERTa—giving SapXLMR-large-5lang and SapXLMR-base-5lang models, respectively. This procedure is similar to that followed by Liu and collaborators [17] to train the original multilingual SapBERT, except that they did not use the several languages from UMLS to pretrain the model and, unlike our translation-based approach, they fine-tuned their models for cross-lingual adaptation.

The key advantages of the approach presented in this paper include a reduction in computational requirements, as there is no need to train a full bi-encoder for each specific language, while still surpassing the performance of such resource-intensive models. Additionally, this methodology enables the exploration of much larger batch sizes, allowing the model to identify similarities between terms within the same batch, thereby achieving earlier and more optimal training convergence.

## Results and discussion

In Subtask 1, SymptomNER, the goal was to detect and recognize named entities within a clinical domain from a series of medical texts. We proposed five models that exhibited the best performance for the validation set, selecting three of them—XLM-R-Galén, BSC-Bio-es, and RoBERTa-Base-Biomedical-es—as individual models for evaluation on the test set, along with two ensembles: one comprising the five models with the highest F1-scores (we named this ensemble icb-uma-ensemble-1), and the other including the three aforementioned models (ensemble icb-uma-ensemble-2). Table 3 shows the results achieved. As detailed in our preliminary work in [26] we set a new benchmark in terms of precision and F1-score for both ensembles 1 and 2, achieving 0.748, 0.804, and 0.699 with ensemble 1, and 0.746, 0.790, and 0.707 with ensemble 2, in precision, recall, and F1-score, respectively. We also maintained very similar results across both the validation and test sets. However, it is not just the ensemble models that have demonstrated high performance; BSC-Bio-es and RoBERTa-Base-Biomedical-es, individually, have shown very competitive results, achieving F1-scores of 0.729 and 0.716, respectively.

**Table 3.** Comparative performance metrics (F1 Score, Precision, and Recall) of individual models and ensembles for the SymptomNER task on validation and test sets

	Validation set (mean ± std)			Test set		
Model name	F1	Precision	Recall	F1	Precision	Recall
BSC-Bio-es	0.723 ± 0.002	0.742 ± 0.003	0.705 ± 0.005	0.729	0.744	0.**713**
Roberta-Base-Biomedical-es	0.720 ± 0.001	0.735 ± 0.002	0.705 ± 0.005	0.716	0.729	0.704
XLM-R-Galén	0.688 ± 0.002	0.728 ± 0.002	0.653 ± 0.005	0.694	0.714	0.682
mBERT-Galén	0.681 ± 0.003	0.715 ± 0.003	0.650 ± 0.006	0.673	0.694	0.652
Beto	0.681 ± 0.002	0.703 ± 0.002	0.661 ± 0.005	0.700	0.711	0.685
icb-uma-ensemble-1	0.744 ± 0.003	0.813 ± 0.002	0.686 ± 0.005	**0.748**	0.**804**	0.699
icb-uma-ensemble-2	0.743 ± 0.002	0.795 ± 0.003	0.697 ± 0.005	0.746	0.790	0.707

Ensemble results set new benchmarks in precision and F1-score, with detailed mean and standard deviation values highlighting the models’ consistency and effectiveness in clinical named entity recognition. The highest-performing model is marked in bold and the second-best underlined.

In Subtask 2, SymptomNorm, the primary goal was to link medical entities from clinical corpora in Spanish to an ontological standard such as SNOMED CT. To accurately assess the developed models, we employed the top-k accuracy metric, varying the value of k between 1, 5, 25, 50, 100, and 200. This approach allowed us to examine the models’ performance across different scenarios, while always prioritizing the top-1 accuracy. The complexity of this task, where the annotated dataset is scarce and the possible codes are significantly numerous, leads us to consider scenarios in which not just one code is provided per mention, but a set of 5–20. In this way, annotation becomes a semi-automatic process rather than a manual one. Our model supplies a narrowed-down subset of candidates, from which the most suitable one is selected.

In this paper, we have focused on the comparison of three different bi-encoders to deal with this NEL task: SapBERT-multilingual, SapXLMR-base-5lang, and SapXLMR-large-5lang. The first one is the original multilingual SapBERT proposed by Liu and collaborators [17]—which currently sets the state of the art for SymptomNorm corpus [5]—trained with the entire UMLS in the form of triplets and subsequently with cross-lingual adjustments. On the other hand, both SapXLMR-large-5lang and SapXLMR-base-5lang were trained by us using triplets from the UMLS, but only selecting the five most prevalent languages relevant to this task. We also compare these three bi-encoders when they are enhanced by the subsequent application of a linear WNT-softmax classification layer to the output of the models (we use the prefix “WNT-” in Table 4 for these resulting models).

**Table 4.** Performance across top-k-accuracy for three bi-encoder models and their counterparts fine-tuned with WNT-softmax, across different k values in Subtask 2, SymptomNorm, evaluated on validation and test datasets

	Validation set						Test set					
Model	@1	@5	@25	@50	@100	@200	@1	@5	@25	@50	@100	@200
SapBERT-multilingual	*0.659*	*0.829*	*0.924*	*0.941*	*0.952*	*0.962*	*0.549*	*0.721*	***0.808***	***0.842***	***0.864***	***0.885***
SapXLMR-large-5lang	0.657	0.829	0.924	0.941	0.952	0.962	0.528	0.693	0.777	0.810	0.832	0.852
SapXLMR-base-5lang	0.657	0.817	0.912	0.928	0.943	0.947	0.543	0.709	0.795	0.823	0.852	0.877
WNT-SapBERT-multilingual	0.717	0.878	0.931	0.943	0.952	0.959	**0.579**	**0.726**	0.801	0.823	0.852	0.871
WNT-SapXLMR-large-5lang	**0.729**	**0.884**	**0.940**	**0.951**	**0.965**	**0.971**	0.551	0.691	0.773	0.813	0.839	0.864
WNT-SapXLMR-base-5lang	0.724	0.872	0.931	0.947	0.957	0.962	0.555	0.709	0.787	0.815	0.839	0.869

The highest-performing model for each k is marked in bold, with the second-best underlined and with italics the current benchmark. (Note: Values represent the average of three runs; standard deviation is omitted as it is approximately 1e-3, owing to adjustments made solely to the WNT-softmax).

From Table 4, it is evident that the bi-encoders alone (top three rows of the table) exhibit robust performance across all top-k accuracy metrics. However, they are almost always surpassed in the lower top-k metrics—i.e. top-1 and top-5—by the classification strategies given by the WNT-softmax-enhanced versions of the models (three lower rows of the table). Thus, the WNT-SapBERT-multilingual achieves 0.579 and 0.726, compared to the 0.549 and 0.721 rates given by the SapBERT-multilingual bi-encoder alone. It is also important to highlight how the application of cross-lingual adjustments made by SapBERT’s authors to get multilingual SapBERT has shown superior performance compared to the two models that we trained specifically with the languages relevant to this task. Moreover, our bi-encoders, i.e. SapXLMR-large-5lang and SapXLMR-base-5lang, still show room for performance improvement, as our limitations of computational resources have impaired them to be trained for more than 1 and 3 epochs, respectively. It would be interesting to explore more intensive training of these models in future research to estimate their full potential.

**Figure 3.**
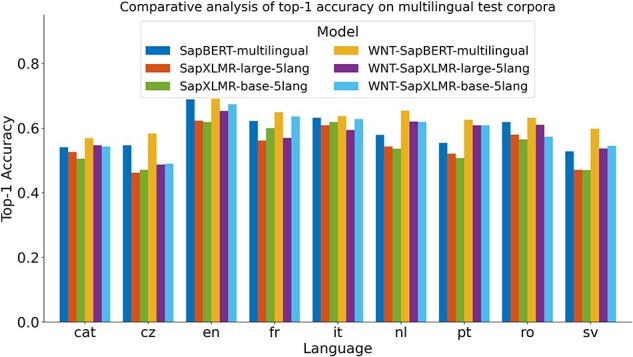
Top-1 accuracy of six models across nine languages: three bi-encoder models and their counterparts with fine-tuned WNT-softmax classification layers.

**Figure 4.**
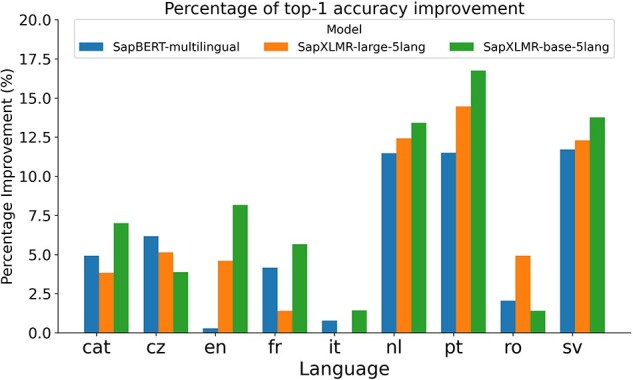
Bar plot of the percentage of increase in top-1 accuracy for each of three bi-encoder models after being supplemented with a fine-tuned WNT-softmax layer to tackle a multilingual NEL task in nine languages.

For Subtask 3, encompassing the normalization of medical entities from multilingual terms, we explored the same approach as applied to SymptomNorm subtask, analyzing the three selected models both as bi-encoders alone and following a classification approach with the WNT-softmax. In Fig. 3, one can examine how the fine-tuning of the WNT-softmax significantly improves the bi-encoder-alone models in top-1-accuracy, the metric chosen by the organizers of this dataset, showcasing superior individual performance compared to the original model. Furthermore, it is noticeable how the difference increases based on how relevant the language is in the UMLS, with the largest disparities seen in Dutch and Swedish. Another observable trend is how our models trained from XLM-RoBERTa—i.e. SapXLMR-large-5lang and SapXLMR-base-5lang—exhibit a higher improvement potential, primarily because they were not as well-adapted to the domain as SapBERT-multilingual was. This is even more apparent in Fig. 4, which shows how much each model has improved after applying the WNT-softmax for each of the languages, with WNT-SapXLMR-base-5lang standing out in Portuguese and Dutch with a difference of 102 points and 75 points in top-1 accuracy, respectively. The performance rates of each model, both the bi-encoders alone and the WNT-softmax-enhanced versions for each language, are shown in detail in Table 5.

**Table 5.** Performance of models in top-1-accuracy for Subtask 3, SymptomMultiNorm, with the best-performing model for each language highlighted in bold, with the second-best underlined and with italics for the current benchmark

Model	cat	cz	en	fr	it	nl	pt	ro	sv
SapBERT-multilingual	*0.541*	*0.547*	*0.689*	*0.622*	*0.632*	*0.579*	*0.554*	*0.619*	*0.528*
SapXLMR-large-5lang	0.526	0.462	0.623	0.562	0.609	0.543	0.521	0.58	0.471
SapXLMR-base-5lang	0.505	0.471	0.619	0.6	0.619	0.536	0.507	0.565	0.47
WNT-SapBERT-multilingual	**0.569**	**0.583**	**0.691**	**0.649**	**0.637**	**0.654**	**0.626**	**0.632**	**0.598**
WNT-SapXLMR-large-5lang	0.547	0.487	0.653	0.57	0.594	0.62	0.609	0.61	0.537
WNT-SapXLMR-base-5lang	0.543	0.49	0.674	0.636	0.628	0.619	0.609	0.573	0.545

Values represent the average of three runs; standard deviation is omitted as it is approximately 1e-3, owing to adjustments made solely to the WNT-softmax. The best-performing model for each language highlighted in bold, with the second-best underlined and with italics for the current benchmark

### Performance analysis by Semantic Tag

For a detailed performance analysis of the models presented in this paper, we focused on the three most frequent semantic tags in the SympTEMIST test set: “finding,” “disorder,” and “morphologic abnormality.” Additionally, we evaluated the accuracy at 1 for Spanish, English, and Swedish. These languages were chosen arbitrarily, with Spanish and English being the two most dominant languages and Swedish being a less common language where our model performed well.

Table 6 demonstrates that the WNT-softmax-multilingual model consistently outperforms the other models across all languages and tags. This model exhibits robustness and adaptability by effectively handling linguistic variations. However, it is noteworthy that while this model excels in most areas, it shows a significant decrease in performance for the “morphologic abnormality” tag in Swedish, indicating a potential area for future improvement.

**Table 6.** Performance comparison of top-1 accuracy for the three most prevalent semantic tags in the test set: “finding” (find), “disorder” (dis), and “morphologic abnormality” (morph), across three languages: Spanish, English, and Swedish

	Spanish			English			Swedish		
Model	find	dis	morph	find	dis	morph	find	dis	morph
WNT-softmax-multilingual	0.570	**0.508**	0.574	**0.694**	**0.634**	0.600	**0.603**	**526**	0.344
SapBERT-multilingual	0.563	0.445	0.529	0.669	0.583	0.556	0.488	0.500	0.406
SapXLMR-large-5lang	0.527	0.387	0.471	0.593	0.566	0.511	0.443	0.415	0.375
SapXLMR-base-5lang	**0.576**	0.463	**0.603**	0.614	0.592	**0.689**	0.489	0.389	**0.438**

The highest-performing model for each language and tag is marked in bold and the second-best is underlined.

On the other hand, the SapBERT-multilingual model achieves competitive performance, particularly in Spanish and English. However, its performance declines noticeably in Swedish. This suggests that while the model is effective in multilingual settings, it may struggle with languages that were underrepresented in the training data.

Finally, the SapXLM-R-large and SapXLM-R-base-5lang models exhibit the least consistent performance across all metrics. These models seem to have particular difficulty with the “disorder” tag in all languages, and their performance in Swedish is significantly lower than in Spanish and English. This inconsistency could be attributed to the models’ inability to generalize effectively across different languages and semantic tags.

Our performance analysis by semantic tag provides valuable insights into the efficacy of our models, highlighting their strengths and weaknesses. These findings will guide our future efforts as we strive to enhance the effectiveness and adaptability of our models in multilingual semantic tagging tasks.

## Conclusions

In this study, we have addressed the tasks of recognizing and linking medical entities in clinical texts, covering the entire pipeline. Our approach unfolds into the three subtasks set forth by the organizers of the SympTEMIST shared task. Thus, for Subtask 1, SymptomNER, our methodology involved the application of Transformer-based multilingual model ensembles, emphasizing the significance of BERT and its variants in NER within the clinical domain. The results demonstrate a significant improvement in precision and F1-score for identifying mentions of signs and symptoms in clinical texts in Spanish.

For the SymptomNorm subtask, we adopted an approach that combined embedding similarity-based models with self-alignment pretraining and in-domain adaptation, derived from multilingual BERT transformers, with a classification-based approach, achieving high top-k accuracy for low k values, such as k = 1 and k = 5. With this novel approach, which combines an information retrieval strategy using bi-encoders with a classification model to tackle NEL tasks, we not only improve the efficiency of the original bi-encoders but also contribute with models that require less computational resources to be trained in multilingual environments.

For the subtask of multilingual medical entity normalization, SymptomMultiNorm, our methodology—based on multilingual bi-encoders followed by a classification layer fine-tuned with the entries obtained from an in-domain KB automatically translated into the target languages—excelled with outstanding effectiveness, surpassing SapBERT-based models in most of the languages analyzed. This underlines the ability of our method to adapt to diverse linguistic contexts and cope with the scarcity of annotated data in less common languages, which is crucial since we did not have access to the KBs in different languages and had to resort to machine translation with generative LLMs.

Thus, our research significantly advances NLP in the clinical domain, not only in entity recognition and linking in medical texts in Spanish but also in a multilingual context. This opens a wide range of practical implications, highlighting approaches that support more efficient clinical decision-making based on a complete NER + NEL pipeline. Additionally, we would like to validate the efficacy of machine translation in clinical texts, as it has only been tested on general domain texts, which may pose a limitation to our work.

For future work, we aim to continue exploring and enhancing NLP techniques in the medical domain to tackle challenges like semantic ambiguity and linguistic variability. Potential directions include leveraging context in entity linking, addressing multiple mentions within the same context to extract relationships between them, and exploiting generative large language models for tasks such as NER or NEL.
